# Identifying the Hotspots on the Top Faces of WD40-Repeat Proteins from Their Primary Sequences by β-Bulges and DHSW Tetrads

**DOI:** 10.1371/journal.pone.0043005

**Published:** 2012-08-15

**Authors:** Xian-Hui Wu, Yang Wang, Zhu Zhuo, Fan Jiang, Yun-Dong Wu

**Affiliations:** 1 Lab of Computational Chemistry and Drug Design, Laboratory of Chemical Genomics, Peking University Shenzhen Graduate School, Shenzhen, People’s Republic of China; 2 College of Chemistry, Peking University, Beijing, People’s Republic of China; Indian Institute of Science, India

## Abstract

The analysis of 36 available crystal structures of WD40 repeat proteins reveals widespread existence of a beta-bulge formed at the beginning of strand a and the end of strand b, termed as WD_b–a_ bulge: among a total of 259 WD40 blades, there are 243 such β-bulges. The R_1_ positions in these WD_b–a_ bulges have fair distributions of Arg, His, Ile, Leu, Lys, Met, Phe, Trp, Tyr and Val residues. These residues protrude on the top face of the WD40 proteins and can serve as hotspots for protein-protein interactions. An analysis of 29 protein complexes formed by 17 WD proteins reveals that these R_1_ residues, along with two other residues (R_1_-2 and D-1), are indeed widely involved in protein-protein interactions. Interestingly, these WD_b–a_ bulges can be easily identified by the 4-amino acid sequences of (V, L, I), R_1_, R_2_, (V, L, I), along with some other significant amino acids. Thus, the hotspots of WD40 proteins on the top face can be readily predicted based on the primary sequences of the proteins. The literature-reported mutagenesis studies for Met30, MDV1, Tup11, COP1 and SPA1, which crystal structures are not available, can be readily understood based on the feature-based method. Applying the method, the twelve potential hotspots on the top face of Tup11 from *S. japonicas* have been identified. Our ITC measurements confirm seven of them, Tyr382, Arg284, Tyr426, Tyr508, Leu559, Lys575 and Ile601, are essential for recognizing Fep1. The ITC measurements further convinced that the feature-based method provides accurate prediction of hotspots on the top face.

## Introduction

WD40-repeat proteins belong to a subfamily of β-propeller proteins [1], [2]. It is one of the largest protein families from yeast to human, consisting about 1% total cell proteins [3]. These proteins show their biofunctions mainly by binding other proteins to form protein complexes [2], [3], [4], which have been found to play important roles in multiple cellular processes. For example, WDR5, EED, RbBP4, RbBP5 and RbBP7 are components of methyltransferases [5], [6]. A couple of WD40 proteins are core components of Cullin-Ring E3 ligases, which play important roles in protein degradation by catalyzing the polyubiqitination [7], [8], [9], [10]. Additionally, WD40 proteins are critical in forming the apoptosome [11], extracellular signal transduction [12], [13], [14], as well as components of the nuclear core [15], [16] and membrane vesicle [17].

**Figure 1:**
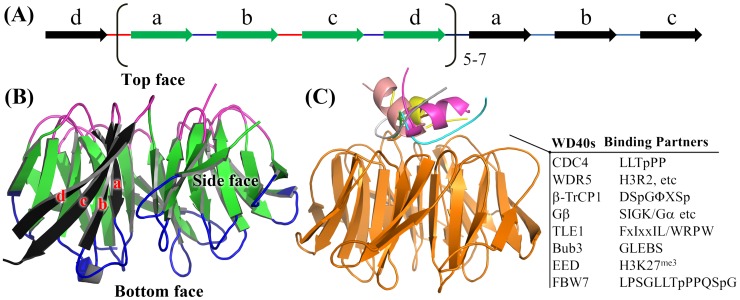
The definition of WD40 blade and binding faces of WD40-repeat protein. (**a**) In sequence order, every WD40 repeat protein is composed of strand d–a–b–c. Structurally, the first strand d and the last strand a–b–c form a four-strand anti-paralleled β-sheet, a WD40 blade, which encloses β-propeller structure. The other WD40 blades are formed by sequential strand a–b–c–d. (**b**) WD40 proteins have top, side and bottom faces. Top face: loops connecting strand b–c and strand d–a; Bottom face: loops connecting strand a–b and strand c–d; Side face: the grooves between two neighbored WD40 blades. (**c**) A majority of interactions are focused on the top faces.

WD40-repeat proteins typically contain 6–8 repeated units in the sequence order. These repeat units fold into 6–8 WD40 blades and form β-propeller structures. According to previous definition, every WD40 repeat folds into strand d–a–b–c [2]. However, strand d and strand a–b–c in a WD40 repeat belong to two different WD40 blades. To avoid the inconsistency between WD40 repeat and WD40 blade, we use WD40 blade as a repeated unit in following description. As shown in Figure 1a, the first strand d at the beginning and the last strand a, b and c at the end form a special WD40 blade, which encloses β-propeller structure. The other WD40 blades are formed by the sequential strand a, b, c and d. Figure 1b shows the folded β-propeller has a top, side and bottom faces. The top face is composed by the loop connecting strand b and c as well as several residues at the beginning of strand a. The bottom face contains two loops, which connects strand a and b, strand c and strand d. There are grooves between two neighbored WD40 blades, which is the side face. Three faces are able to address proteins to form protein complexes. Figure 1c gives some examples of the well-studied interactions involved by WD40 proteins, which predominantly occur on their top faces. WDR5 and EED bind H3R2 and tri-methylated H3K27, respectively, by cation-π interaction [18], [19]. FBW7 and β-TrCP, as an adaptor of SCF complex, recognize phosphorylated CycE and β-catenin, respectively, and facilitate their poly-ubiquitination [20], [21]. Gβ [22], [23] and TLE1 [24], [25] can bind multiple proteins on the top face by the hydrophobic and hydrophilic interactions. Besides forming complex with protein, DDB2 also binds DNA on its top face [26]. Gemin5 was proposed to interact with RNA on the top face as well [27]. In order to understand the interaction, the critical residues need to be identified on the top face. If these key residues could be predicted based on the primary sequence without structural information on the protein complexes, it will provide substantial help for addressing the protein interactions on the top faces of WD40 proteins.

**Figure 2:**
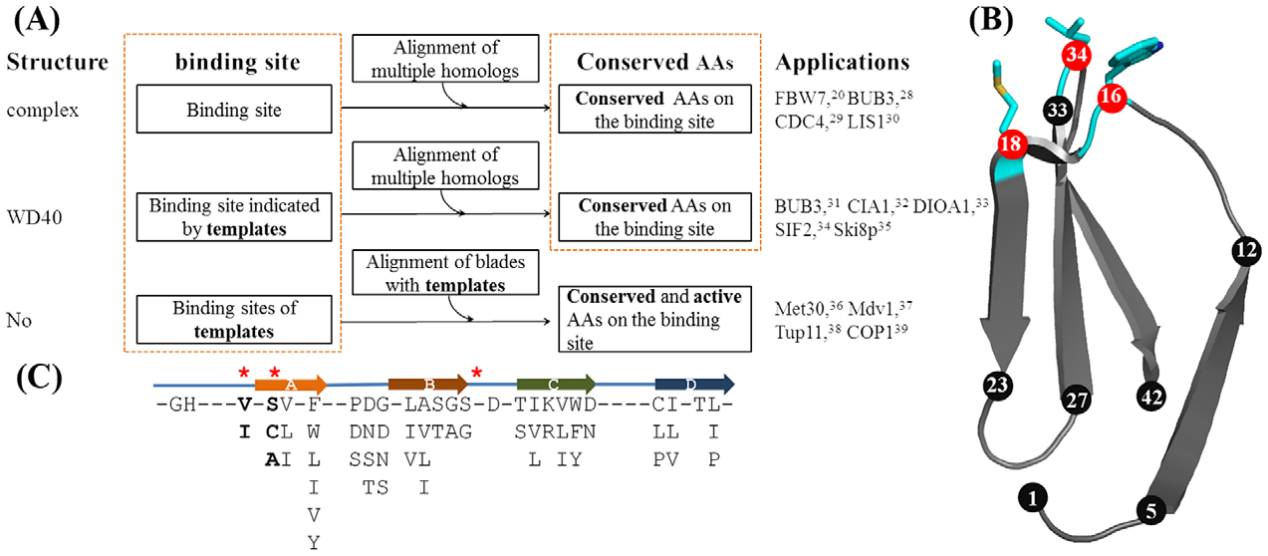
Empirical methods for identifying hotspots on the top faces of WD40 proteins. (**a**). Identifying hotspots by two sequential steps: 1. Determining loops composed of top faces; 2. Choosing the functional residues on two loops. (**b**). The 16^th^, 18^th^ and 34^th^ residues are repeatedly involved in the protein-protein interaction. Here, strand d–a–b–c was presented in the sequence order. (**c**). Three hotspots proposed by Stirnimann *et al.* ^3^ (marked by red-stars) are incorrectly mapped on the common sequence pattern.

However, the currently available methods highly depend on the availability of crystal structures and accumulated experimental observations. Figure 2a shows the widely utilized method in determining the hotspots residues of WD40 proteins. This method requires two sequential steps. The first step is to determine loops connecting strand b–c and strand d–a. At the second step, hotspots are further identified from loops. There are three cases in practice. In every situation, both steps have a bit difference. In the first case, if there is a crystal structure of protein complex, the critical residues for interaction on the loop d–a and loop b–c are obvious [20], [28], [29], [30]. In the second case, if only the crystal structure of WD40 protein is available, the necessary residues for interaction at two loops are defined by comparing with the crystal structures of well-studied WD40 proteins, such as Gβ and TUP1 [31], [32], [33], [34], [35]. After that, conserved residues are chosen for experimental tests by aligning multiple homologs across different species. In the last case of no crystal structure, several well-studied WD40 proteins are acquired as templates, which crystal structures have already been determined. To obtain the loop d–a and loop b–c, the predicted WD40 blades are aligned with those in the templates. Then, the residues in two loops are selectively studied if the corresponding residues in the templates are already proved to be indispensable for protein-protein [36], [37], [38], [39].

As mentioned above, the empirical method highly depends on the crystal structures of protein complexes. In the case that WD40 proteins have no crystal structures, the empirical method can’t provide efficient resolution, such as Tup11 from *S. pombe*. Even though it has ∼53% sequence identity to a well-studied template TUP1, only two residues, Tyr362 and Leu542, are identified to be essential for Fep1 interaction [38]. Apparently, the interaction needs more residues. Even though WD40 protein itself has been crystallized, they still can’t provide much useful information for identifying key residues in protein-protein interaction. For example, although the resolution of CIA1 crystal structure is as high as 1.7Å, it indicates little information of interaction on the top face. By utilizing Gβ and Tup1 as templates, only one residue, Arg127, was found to be important for recognizing protein after screening six potential residues on the top face [32].

In 1996, Gaudet *et al.* reported the crystal structure of G_β_-phosducin complex [13]. As shown in Figure 2b, they noticed that the 16^th^, 18^th^ and 34^th^ residues of each WD40 blade in G_β_ can be involved in binding Phosducin on the top face. They extrude on the top face of G_β_ protein. The 16^th^ and 18^th^ residues are close to the beginning of strand a and the 34^th^ residue locates right before the conserved Asp in G_β_. Later, in the case of β-TrCP1-β-catenin complex, the 16^th^ and 18^th^ residues are described to be at the beginning of strand a based on its crystal structure. Tyr271, Leu474 and Arg474 at the beginnings of two strand a are further proved to be indispensable for binding β-catenin by mutagenesis studies [21]. However, this rule has found few applications in the literature, probably due to the following reasons: (1) the precise positions of 16^th^, 18^th^, and 34^th^ residues can’t be applied directly to other WD40 proteins because the lengths of WD40 blades are variable. (2) The beginning of strand a is irregular and hardly defined precisely. Recently, an analysis of peptide-WD protein complexes by Stirnimann *et al.* [3] revealed that such three residues are generally required for recognizing peptides on the top face by WD40 proteins. In addition, the 16^th^, 18^th^ and 34^th^ residues are pointed out on the structural elements proposed by Smith *et al.* [4] as shown in Figure 2c. However, the proposed 16^th^ and 18^th^ residues usually point inward as the 3D structure was taken into the consideration, which is unavailable in protein-protein interaction.

In this manuscript, we analyzed 36 WD40-repeat proteins, which crystal structures are available [40]. Based on the survey of the available crystallographic and mutagenesis studies, we will pursue the hypothesis that residues at specific positions are responsible for the protein-protein interactions on the top face of the WD40 domains partly based on the structural elements [4]. We will present a general prediction method for the identification of these key residues using only the sequence information. Finally, this method will be validated using the results of mutagenesis studies and ITC measurements.

## Results and Discussion

### β-bulges in WD40 Blades

**Figure 3:**
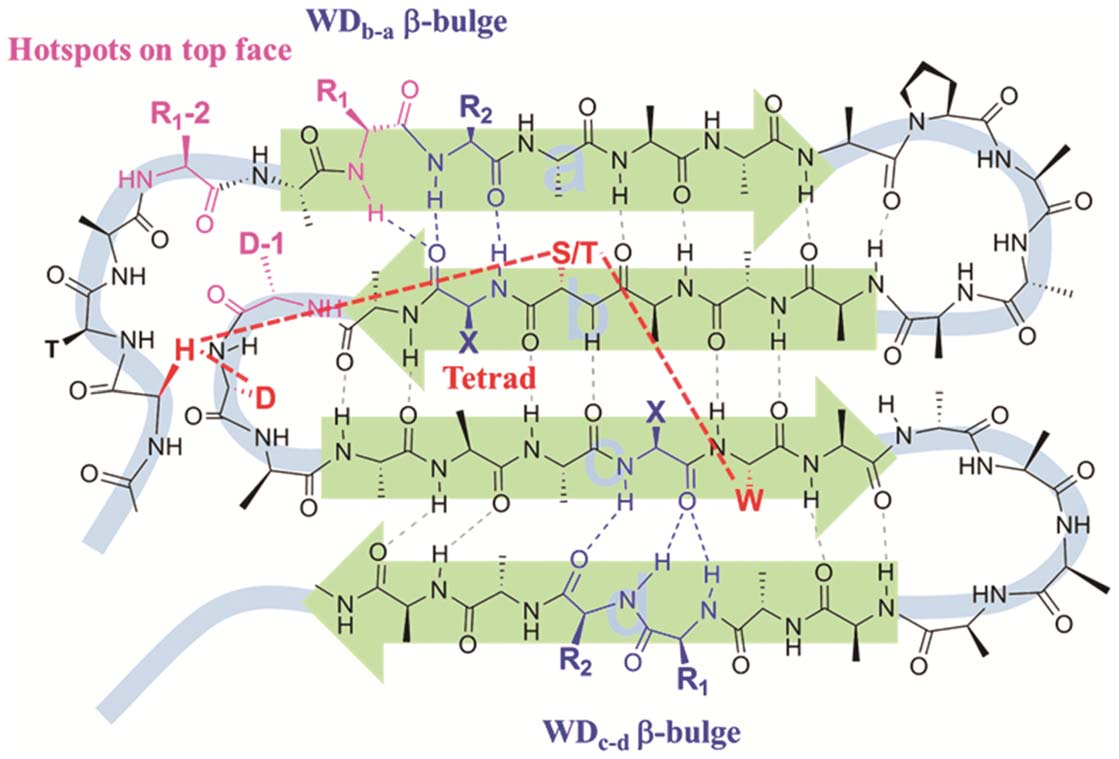
The hydrogen bonds and structural features in the general WD40 blade by strand a–b–c–d. Although the enclosed WD40 blade has the different order in sequence, their hydrogen bonds, β-bulges and DHSW tetrad are in common. the DHSW tetrad: Asp-His-Ser/Thr-Trp (in red). WD_b–a_ β-bulge: formed in strand b and a; WD_c–d_ β-bulges: formed in strand c and d. X, R_1_, R_2_ (in blue). Three residues, R_1_-2, R_1_ and D-1 colored in pink are highlighted here for the following discussion of interaction on the top face. R_1_ as a binding residue is “R_1_ residue” of WD_b–a_ bulge. R_1_-2 locates on two residues before R_1_ of WD_b–a_. D-1 locates before Asp of DHSW tetrad.

An analysis of 36 available WD proteins with crystal structures has been carried out. Totally, there are 259 WD40 blades and 133 of them have DHSW hydrogen-bonded tetrads. As depicted in **Figure 3**, 243 WD40 blades have a WD_b–a_ β-bulge, and 206 WD40 blades have a WD_c–d_ bulge. Interestingly, all WD40 blades, which contain the DHSW tetrads, have WD_b–a_ bulges.

β-bulge is a special structure in β-sheet, which includes two residues (R_1_, R_2_) in one strand H-bonded to a single residue (X) in the opposite strand. Structurally they cause the change of direction for a β-sheet [41], [42]. They disrupt further extension of β-sheet and therefore, have been proposed to be naturally designed structure to prevent aggregation of β-sheet-rich proteins [43]. Currently available experiments suggest β-bulges play critical roles in the protein folding and maintaining the function in the ubiquitin [44], [45], *E. coli* dihydrofolate reductase [46], bacterial RNase [47] and mammalian defensing [48].

### WD_b–a_ Bulges have Distinct Residue Propensities

**Figure 4:**
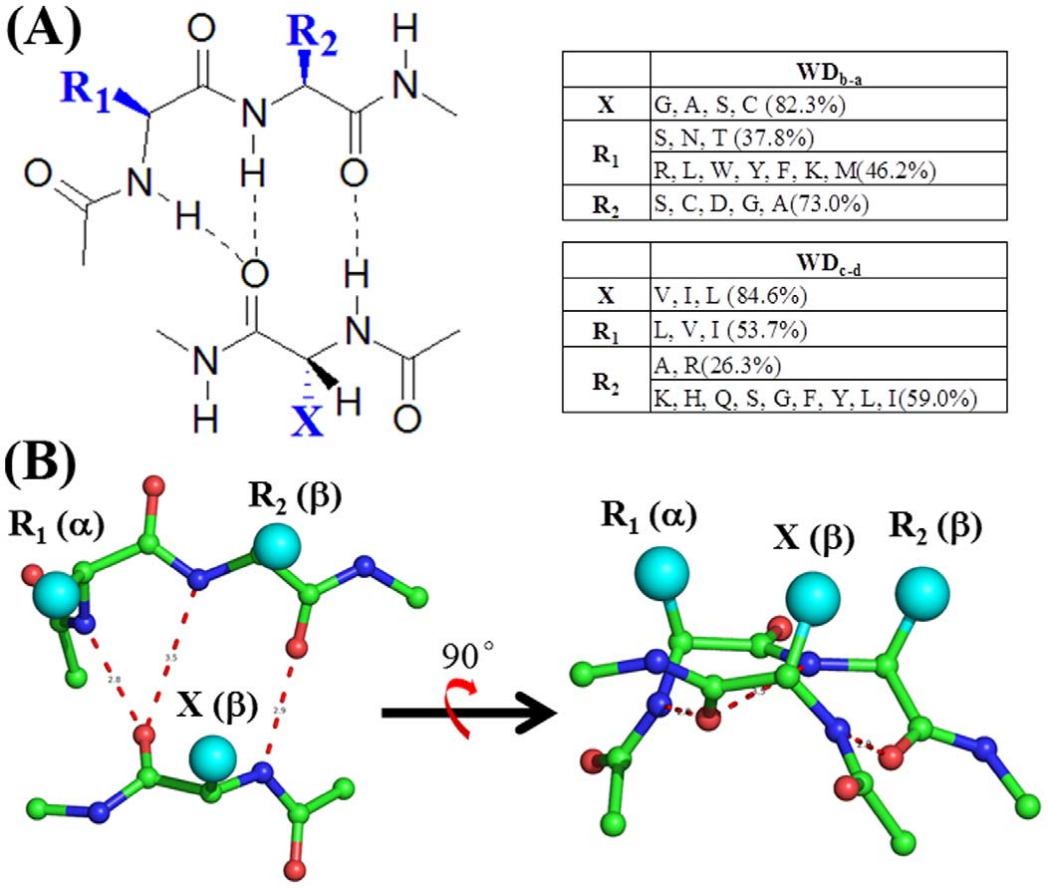
The summation of WD_b–a_ and WD_c–d_ β–bulges. (**a**) The schematic view and residue dominances of WD_b–a_ and WD_c–d_ bulges. (**b**) Two-side view of β-bulge. X, R_1_:α-conformation; R_2_: β-conformation.

**Table 1** summarizes the residue distributions (%) in WD_b–a_ and WD_c–d_ β-bulges. Several characters are apparent:

1. As summarized in Figure 4a, the most significant feature of WD_c–d_ is the dominance of bulky residues Val, Ile and Leu in the X and R_1_ positions, 84.6%, 53.7%, respectively. This situation is similar to that in the classic β-bulge of all proteins [41], [42].
2. Contrast to the WD_c–d_, the X position of the WD_b–a_ is featured with small residues, Gly and Ala in 38.2% and 25.2%, respectively. Together with Ser and Cys, these four types of residues compose of 82.3% of X residues.
3. The most favorable R_1_ residues of the WD_b–a_ are Asn, Ser and Thr, totalling to 37.8%. However, Arg, Glu, Gln, His, Ile, Leu, Lys, Met, Phe, Trp, Tyr and Val all have reasonable populations, totaling to about 57.6%. Thus, R_1_ residues are most diversified.
4. As shown in **Table 1**, WD_b–a_ has significantly increased populations of the hydrogen bond donor/acceptor residues, Asp, Asn, Cys, Ser and Thr with the comparison of WD_c–d_. In particular, Cys is not common in classic β-bulges, but has high populations in the X and R_2_ positions of the WD_b–a_.

**Table 1.The:** percentages of the 20 natural amino acid residues in the X, R_1_ and R_2_ positions of WD_b–a_ and WD_c–d_ β-bulges.

		G	P	A	F	Y	W	H	E	Q	K	R	M	L	C	S	D	N	V	I	T
**WD_b–a_**	**X**	**38.2**	0.0	**25.2**	0.4	0.0	0.0	0.0	0.4	0.0	1.3	0.4	0.8	0.8	9.7	9.2	0.0	0.0	7.6	0.8	5.0
	**R_1_**	1.3	0.0	0.8	6.7	7.1	8.0	2.9	2.1	1.3	3.8	**10.1**	2.5	8.0	2.5	**13.9**	0.8	**13.0**	2.5	1.7	**10.9**
	**R_2_**	7.1	0.0	9.2	1.3	0.4	0.0	3.8	0.4	4.6	2.5	2.5	0.4	1.3	**16.4**	**27.7**	**12.6**	1.3	2.1	0.4	5.9
**WD_c–d_**	**X**	1.0	0.0	2.0	3.5	0.5	0.5	0.0	0.0	1.0	0.0	0.5	1.0	**24.4**	2.0	2.0	1.0	0.0	**31.3**	**28.9**	0.5
	**R_1_**	1.0	0.0	2.5	2.5	1.5	0.0	2.0	6.0	2.0	8.5	4.0	2.5	**20.4**	0.5	4.5	3.5	0.5	**18.9**	**14.4**	5.0
	**R_2_**	6.5	0.0	**15.4**	5.5	4.5	0.5	9.0	2.0	8.5	9.5	**10.9**	2.0	4.5	1.0	7.0	1.5	3.0	2.0	4.0	3.0

Figure 4 summarizes the residue preferences of WD_b–a_ and WD_c–d_ are dramatically different in the X, R_1_ and R_2_ positions. The side chains of X, R_1_ and R_2_ point to the same side and are capable of interaction by the hydrogen-bond or hydrophobic packing. Thus, the different residue preference of WD_b–a_ bulge suggests it play dual roles in the function and structure. In further presentation, we will only focus on the structure and function of WD_b–a_ bulge with its unusual residue propensity.

### Understanding the Unusual Residue Propensity

**Figure 5:**
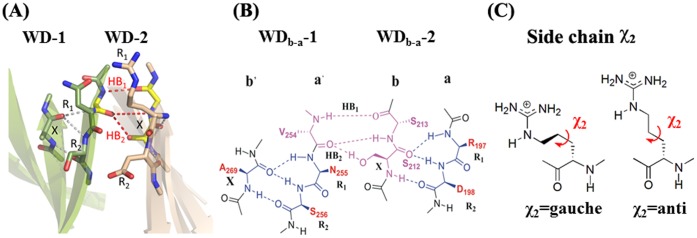
The interaction involved in WD_b–a_ β-bulges between two neighbored WD40 blades. (**a**) and (**b**) show the hydrogen-bonding interactions provided by two WD_b–a_ of neighboring WD40 blades in 3D and 2D, respectively. Those residues composed of WD_b–a_ bulges are shown in stick as numbered in BUB3 (1YFQ). The hydrogen bonds, HB_1_ and HB_2_, are formed inter-blade by Ser213 backbone (X+1 position) and Ser212 (X position) side chain with Val254 (R_1_-1) at the other WD40 blade. (**c**) χ2 dihedral angles of R_1_ residues. As χ2 is at gauche-conformation, Arg points upward. If χ2 is at anti-conformation, Arg point to WD40-1, which is unacceptable due to the crowdedness.

**Figure 5a** shows the topology and WD_b–a_ bulges of two neighboring WD40 blades. For clarity, the loops connecting strands are deleted. **Figure 5b** show the detailed hydrogen bond network formed between the two WD40 blades. The abundant Asp, Asn, Cys, Ser and Thr in the X (23.9%), R_1_ (41.2%) and R_2_ (63.9%) positions, of the WD_b–a_ usually form the inter-blade hydrogen bonds. Besides forming the intro-blade hydrogen bonds in the β-bulges, two inter-blade hydrogen bonds, HB_1_ and HB_2_, are normally formed between the X+1 residue (Ser213) in the WD_b–a_-2 and the R_1_-1 residue (Val254) of WD_b–a_-1. However, if a hydrogen bond donor (Asn, Cys, Ser and Thr) is present at the X, R_1_ or R_2_ position of the WD_b–a_-2, another hydrogen bond is formed between these sidechains and the carbonyl group of the R_1_-1 position of WD_b–a_-1 (**Figure S1**). The rich Asp residues in the R_2_ positions are mainly form the salt-bridges with Arg/Lys from the strand a on the other WD40 blade (**Figure S2**). Therefore, all these unusual residues are able to stabilize entire protein structure by providing interblade interactions.

Our analysis also indicates that in the WD_b–a_, almost half of R_1_ residues with large sidechains as shown in Figure 4 are in an unfavorable conformation. Figure 5c shows χ_2_ dihedral angle of Arg, which usually favors an anti-rotamer, is forced to adapt a gauche rotamer due to the steric crowdedness between the two adjacent WD40 blades. This unfavorable conformation ensures the sidechains to protrude upward and provide highly energetic interaction [49], [50]. Thus, R_1_ residues of WD_b–a_ bulges are likely to be the hotspots if they belong to the aromatic (Phe, Try, Trp), bulky (Ile, Leu, Val) and polar (Arg, Lys, Asp, Glu, Asn and Gln) residues [49], [50], [51]. To confirm the assumption, we carried out a survey of mutagenesis studies of protein complexes, which structures are available.

### R_1_ of WD_b–a_ Along with R_1_-2 and D-1 are Three Residues Generally Required for Recognizing Proteins/peptides on the Top Face of WD40 Proteins

**Figure 6:**
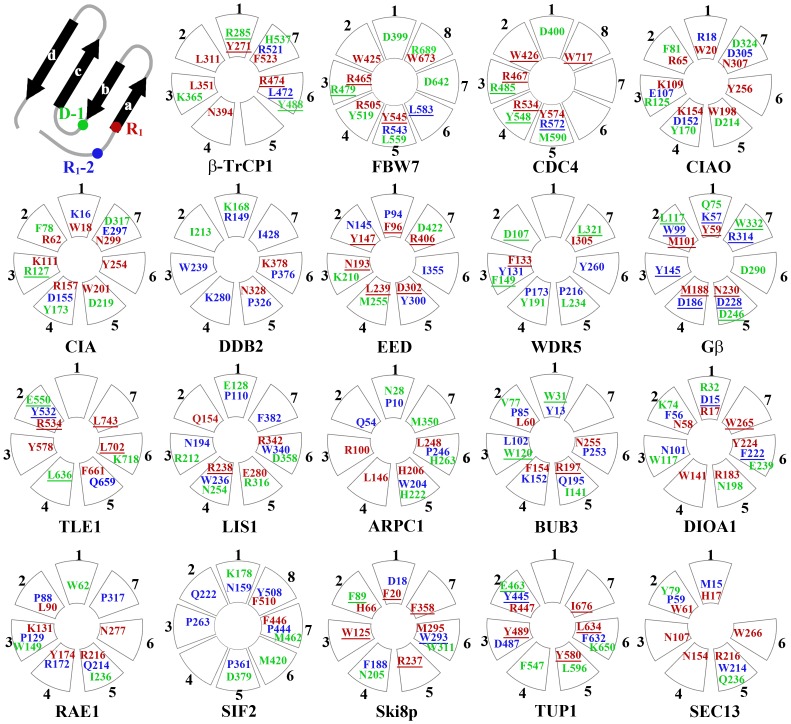
The summation of crystallographic and mutagenesis studies of hotspots on the top faces of 19 representative WD proteins. As depicted on the top left corner, every WD40 blade is composed of four-strand anti-paralleled β-sheet implicitly. R_1_-2, R_1_ and D-1 residues are colored by blue, red and green, respectively. All listed residues are supposed to be important for protein-protein interaction on the top face. Some of them highlighted by the underlines were further convinced by mutagenesis studies.

As shown in **Figure 2b**, we found that R_1_ in the WD_b–a_ bulge is at the 18^th^ residue in Gβ.^15^ The 16^th^ residue locates at two residues before R_1_ (R_1_-2). Meanwhile, the 34^th^ residue locates one residue before Asp (D-1) in the DHSW tetrad. Interestingly, their locations are highly relevant to the positions of structural features in Gβ. Besides Gβ, an analysis of reported WD40-peptides/protein complexes reveals that R_1_-2, R_1_ and D-1 are generally required for interaction. Totally, there are 29 protein complexes formed by 17 WD proteins on the top face besides five complexes formed by FBW7/CDC4/Doa1-ubiquitin [33], Ski8p-Ski3p [35] and Tup1-Metα [52], which only have the crystal structures for WD proteins. By the crystallographic studies, R_1_-2, R_1_ and D-1 are found to be extensively involved in binding proteins/peptides in these protein complexes. Furthermore, some residues are further convinced to be indispensable by the mutagenesis studies, which are highlighted by the underlines as shown in Figure 6. The detailed information of 29 complexes is listed in Table S1. Noticeably, few residues other than R_1_-2, R_1_ and D-1 are essential for the binding.

### R_1_ Residue Might Play More Important Roles in Protein-protein Interaction through the Analysis of Complexes

In some protein complexes, several R_1_ residues are commonly required even in binding different proteins on the top face. Bub3 [28], [31], FBW7 [20], Gβ [12], [13], [14], [22], [23] and WDR5 [18], [53] have been all proved to share R_1_ residues to bind different sequences/proteins. On the other side, one R_1_ residue is enough to define its selectivity. LIS1 and TLE1 interact with different proteins with the use of different R_1_ residues. Both PAF-AFα2 and Nde1 have been found to bind LIS1 on the top face. Mutation of Arg238, a R_1_ residue, to Ala does not prevent the interaction between LIS1 and Nde1 *in vitro*, while it abolishes the interaction between LIS1 and PAF-AFα2 completely [30]. TLE1 can bind both WRPW motif in Hes and FxIxxIL motif in Eh1 on the top face [24]. Leu743, a R_1_ residue as replaced by Phe, selectively eliminates the interaction between TLE1 and WRPW motif and has little effect on TLE1-FxIxxIL interaction [54].

**Figure 7:**
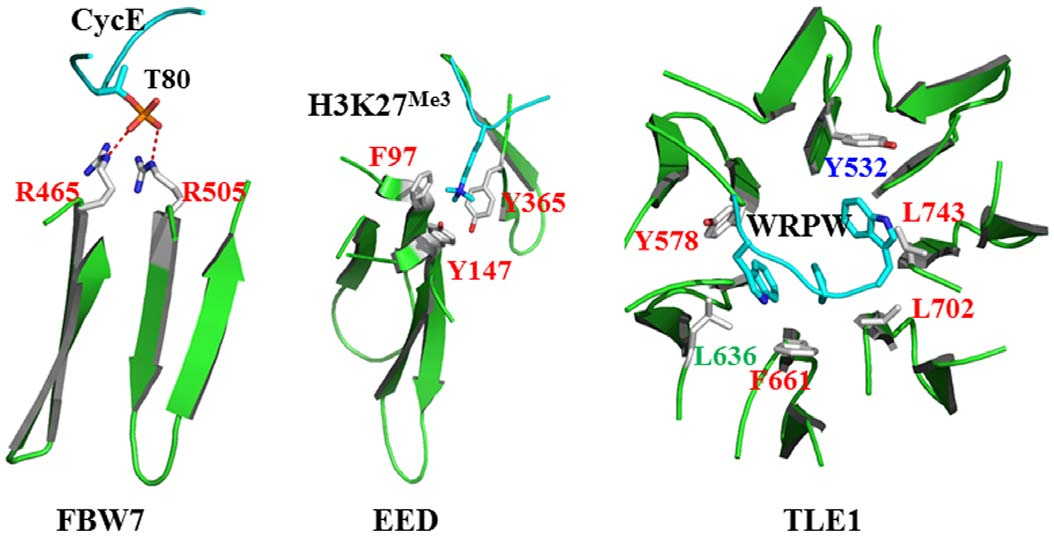
Three representative binding modes provided by WD40 proteins. Binding negatively-charged group: R465 and R505 of two neighboring WD40 blades at FBW7 interact with phosphorylated Thr80 of Cyclin E. **Binding positively-charged group**: EED applies two R_1_, F97 and Y147, as well as Y365 to selectively interact with trimethyl-H3K27. **Interacting hydrophobic group**: Hydrophobic ring formed by a series of residues in TLE1 accommodates WRPW tetrapeptide. R_1_-2, R_1_ and D-1 residues are colored by blue, red and green, respectively.

Expectedly, these residues form a cluster in the protein-protein interaction in a majority of cases although we can’t exclude that Arg, Ile, Leu, Lys, Met, Phe, Trp and Tyr may function as a binding hotspot individually. Charged residues presented at R_1_-2, R_1_ and D-1 of neighboring WD40 blades generate a positively-charged patch to accommodate negatively-charged groups, such as FBW7 and CDC4. Figure 7 shows FBW7 selectively interact with phosphorylated Thr80 of Cyclin E with the use of Arg465 and Arg505. The similar character can be found in CIAO and CIA as shown in Figure 6. We speculate that CIAO and CIA may bind phosphorylated proteins due to their tandem presented Arg and Lys in the R_1_ positions. Several residues including an aromatic sidechain, such as Phe, Tyr and Trp, can form a cluster to accommodate cationic group by the cation-π interactions. Figure 7 shows R_1_ residues Phe96 and Tyr147 in EED are both essential for the recognition of tri-methylated H3K27 through cation-π interaction. But, this kind of binding capability is uneasy to predict because it requires more factors than residue types. Hydrophobic residues located on R_1_-2, R_1_ and D-1 of sequential WD40 blades form a hydrophobic ring and aim to interact hydrophobic residues or patches. Figure 7 presents that Tyr532, Tyr578, Leu636, Phe661, Leu702 and Leu743 in TLE1 (PDB_ID: 1GXR) provide a hydrophobic interaction to bind WPRW. By this means, Figure 6 shows a few WD40 proteins are able to interact with hydrophobic patch, such as ARPC1, BUB3, SIF2 and Ski8p. Because R_1_-2, R_1_ and D-1 as well as organization of these residues can provide critical information for protein-protein interaction on the top face, it’s meaningful to predict these residues precisely from their primary sequences.

### Identifying R_1_-2, R_1_ and D-1 for WD40 Proteins from the Primary Sequence

The structural elements of WD40 blade developed by Smith, *et al.* [4] are extremely helpful for understanding the topology of β-propeller and identifying the predicted surface residues. To provide a general concept, four strands in the WD40 blade share the constant starts and lengths by ignoring their variability. Therefore, the structural element is unsuitable for locating residues accurately on the primary sequence by unclear description of “at the beginning of strand a” and “the end of strand b” as shown in Figure 2c. Encouraged by the relevance of hotspots to the structural features, we developed a feature-based method to predict R_1_-2, R_1_ and D-1 from primary sequences based on β-bulges and DHSW tetrads.

**Figure 8:**
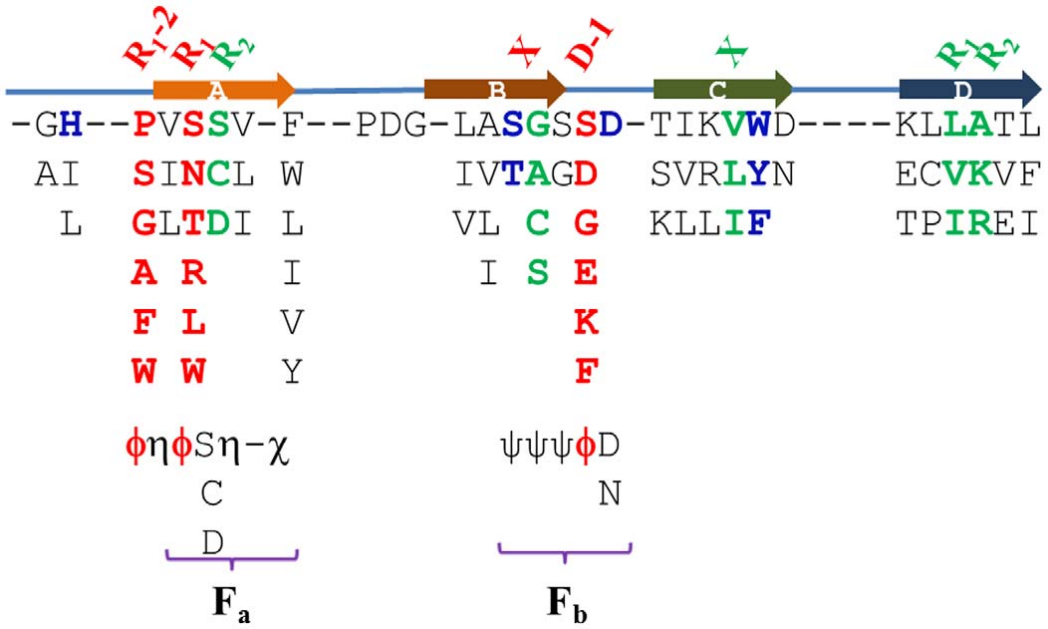
R_1_-2, R_1_ and D-1 are predicted from WD40 blade primary sequence with the use of structural features. The residues of WD40 blade are in the order of frequencies along the vertical line. The blue-colored residues are those in the DHSW tetrad. R_1_, R_2_ and X of β-bulges are highlighted by green color except for R_1_ of WD_b–a_. The red-colored residues, R_1_-2, R_1_ and D-1, are potential hotspots. F_a_ and F_b_ are two fingerprint sequences. η represents bulky residues: Ile, Leu and Val. χ represents Trp, Phe, Tyr, Ile, Leu and Val. ψ represents residues with small side chains: Gly, Ala, Ser, Cys, Thr. φ are potential hotspots.

**Figure 8** shows the common sequence pattern of 259 WD40 blades in 36 WD proteins, which is derived from preferentially aligning the residues in the DHSW tetrad and β-bulges. It has the similar residue frequencies to that provided by Neer *et al.* [2] although the tiny difference can’t be excluded. Due to the structural and biofunctional requirement, the locations of residues in the DHSW [55], [56] and β-bulges are conserved on the primary sequence. The positions of R_1_-2, R_1_ and D-1 are clearly defined at the common sequence pattern on the basis of structural features.

The feature-based method for identifying three residues is divided into three steps: 1. Annotation of WD40 blades. Typically, most of WD40 blades are annotated by SMART [57] or in UniProt [58]. So, we use the annotation in UniProt for consistency. 2. Determining the DHSW tetrad and two fingerprint sequences. 3. Define R_1_-2, R_1_ and D-1 by their dependence. If WD40 blade includes a DHSW tetrad, R_1_-2, R_1_ and D-1 are easily defined according to the relative position of Asp, His, Ser and Trp in the DHSW tetrad [55], [56]. If there is no DHSW tetrad, two fingerprint sequences, F_a_ and F_b_, should be identified in advances within a WD40 blade as shown in **Figure 8**. In F_a_, two η should be Ile, Leu and Val, which enclose R_1_ and R_2_. χ is the end of strand a, which dominants by aromatic or bulky residues. In F_b_, three ψ are those residues with small side chains. Occasionally, Asp/Asn can be replaced by Ser, Thr, Glu or Gln. If WD40 blade contains both F_a_ and F_b_ sequences, R_1_-2 and R_1_ are two φ residues in F_a_, while D-1 is φ residue in F_b_. If R_1_-2, R_1_ and D-1 belong to the aromatic (Phe, Try, Trp), bulky (Ile, Leu, Val) and polar (Arg, Lys, Asp, Glu, Asn and Gln) residues, they could be potential hotspots on the top face.

### The Predicted R_1_ Propensities from 629 Primary Sequences are in Agreement with those Derived from Crystal Structures

**Figure 9:**
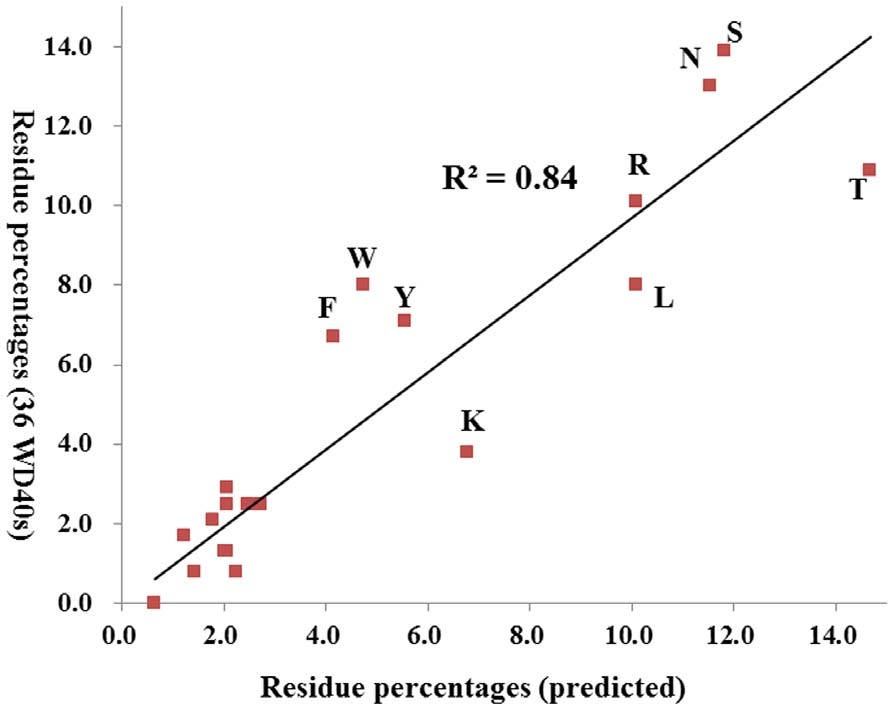
The correlation of R_1_ propensity between x-ray crystal structure and WD40 blade primary sequences. The most dominant residues, Ser, Thr, Asn, Arg, Leu, Trp, Phe, Tyr and Lys are qualitatively consistent.

To test the applicability of these straightforward and simple rules, we carried out prediction for 3939 annotated WD40 blades in UniProt [58] which are composed of 629 WD40 proteins. All these proteins have less than 50% sequence identities. The feature-based method can identify R_1_-2, R_1_ and D-1 residues in 2,200 WD40 blades by DHSW tetrad or two fingerprint sequences. The coverage of prediction is about 55.9%. Figure 9 presents the correlated percentage of predicted R_1_ residue to those in 36 WD40 proteins. R^2^ value is about 0.84. The most dominant residues at R_1_ position have quite similar tendency, such as Ser, Thr and Asn. In addition, the other residues, Arg, Leu, Try, Tyr, Phe and Lys, also have the similar percentages, which are popular as hotspots. Statistically, the feature-based method can predict hotspots on the top face quite well. As we incorporated the residue propensity and structural features into prediction quantitatively, the coverage of prediction reaches around 95%. The responding R^2^ value is also much better (unpublished data).

### The Predicted R_1_-2, R_1_ and D-1 are Consistent with Mutagenesis Studies of WD40 Proteins

Besides statistical evaluation, we further picked out all WD proteins from the test database (629 proteins in UniProt), which structures have not been determined, but mutagenesis studies of predicted R_1_-2, R_1_ and D-1 residues have been reported. A total of six R_1_, seven R_1_-2, four D-1, two X and one R_1_-3 mutations were found in Met30(*S. cerevisiae*) [36], MDV1(*S. cerevisiae*) [37], Tup11(*S. pombe*) [38], COP1(*Arabidopsis*) [39] and SPA1(*Arabidopsis*) [59].

**Figure 10:**
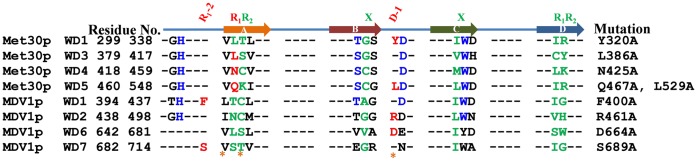
The consistency of predicted R_1_, R_1_-2 and D-1 in WD40 blades of Met30p and MDV1p with the mutagenesis studies. The blue-colored residues are in the predicted DHSW tetrad. The green-colored residues are in the predicted WD_b–a_ and WD_c–d_ bulges. The red-colored residues were studied by the mutagenesis. The last WD40 blade is composed of strand d at the beginning of WD40 domain and the last strand a, b and c.

**Figure 10** shows the Ala substitutions for Met30 and MDV1 for the predicted R_1_-2, R_1_ and D-1. As for Met30, eleven residues were chosen on the basis of consensus sequence pattern [2], [4] and the structural alignment with β-TrCP [21] as well as CDC4 [29]. Substituted by Ala, five residues are found to be important for interacting with Met4 [36]. Compared with our predicted result, three of them are R_1_ residues (Leu386, Asn425, Gln467) and two of them are D-1 (Tyr320, Leu529). With the use of Gβ [12] and Tup1 [60] as templates, Phe400, Arg461, Asp664 and Ser689 of MDV1 were replaced by Ala. Only Arg461 was detected to be essential for interacting with Dnm1 [37], which is D-1 according to our prediction. The mutagenesis studies of COP1 [39] and SPA1 [59] are also consistent with our prediction because those residues, which are importance for protein-protein interaction, are at the predicted R_1_-2, R_1_ and D-1 positions. The detailed interpretation of experimental observation is in the supplemental material (**Figure S3**). As a result, the available results support the feature-based method is more efficient at predicting key residues for protein-protein interaction on the top face from the primary sequence.

### The Feature-based Method is Able to Identify R_1_-2, R_1_ and D-1 Residues Precisely and Comprehensively on the Top Face

To demonstrate its ability, we took Tup11-Fep1 interaction for an example. By the prediction, R_1_-2, R_1_ and D-1 of Tup11 *S. japonicus*, a WD40-repeat protein, are identified for interacting with Fep1 from the primary sequence. Tup11 has been chosen as a demonstration by two reasons. 1. It was discovered to repress the transcription of iron-uptake genes *fio1^+^*, *fip1^+^*, *frp1^+^* and *str1^+^* by binding a GATA-factor Fep1. Because eukaryotes are short of the egress system for iron, the interaction between Tup11 and Fep1 is important for understanding the iron homeostasis [61]. 2. Only two residues were identified to be essential for binding Fep1 on the top face with the use of Tup1 as template, which has as high as 53% sequence identities [38].

### The Predicted R_1_-2, R_1_ and D-1 are Substituted by Ala and their Binding Affinities to Fep1 are Measured by ITC

**Figure 11:**
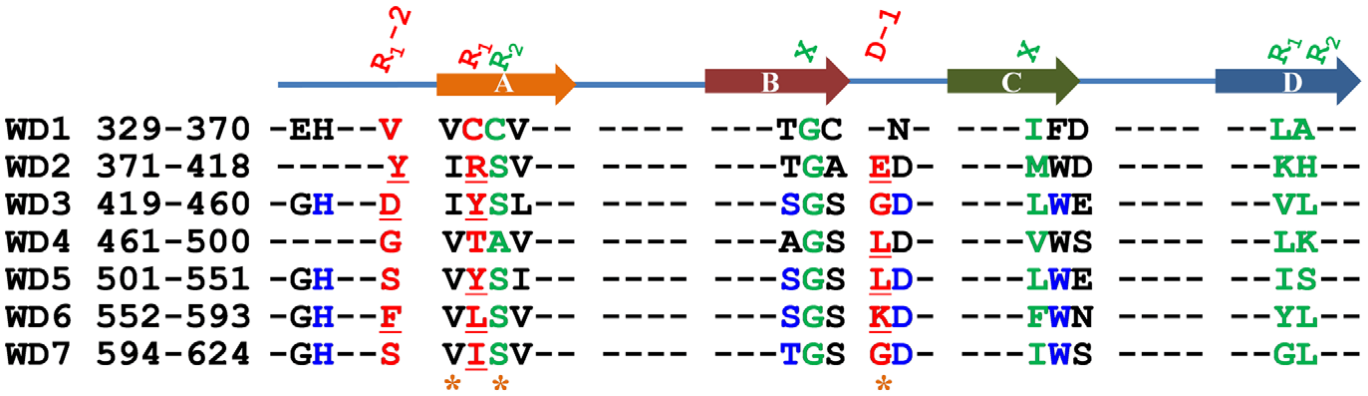
The potential “hotspots” on the top face of Tup11. The predicted R_1_-2, R_1_ and D-1 residues in WD40 blades marked by red-color. The underlined residues besides Cys337 and Thr467 are substituted by Ala or Ser. The last WD40 blade is composed of strand d at the beginning of WD40 domain and the last strand a, b and c.

**Figure 12:**
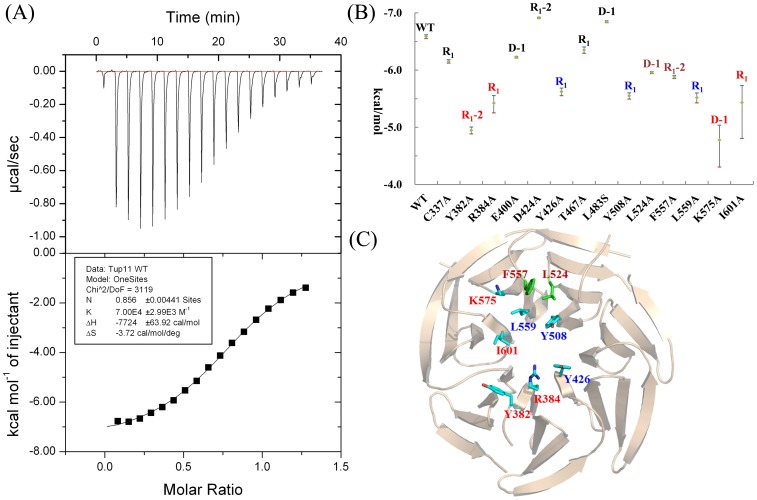
ITC measurements of Fep1 and Tup11 wild type as well as its Ala substitutions. (**a**). The ITC profile of the wild type Tup11-Fep1 interaction. (**b**). The measured binding affinities of the interactions between Tup11 as well as its mutants and Fep1 by ITC. (**c**). The critical residues on top face of Tup11.

As shown in **Figure 11**, a total of 12 underlined residues might be responsible for binding Fep1. Except for Leu384Ser, eleven residues and Cys337 as well as Thr467 are singly substituted by Ala. **Figure 12a** shows *K_d_* of the wild type Tup11-Fep1 interaction, measured by ITC, is about ∼10 µM, corresponding to ∼6.6 kcal/mol for the binding free energy. **Figure 12b** shows the variation of binding affinities by Ala substitutions. As for R_1_ positions, Cyc337 and Thr467 have little effect on binding Fep1 because both residues don’t belong to hotspots. The remained five residues are all critical for binding Fep1. The Ala substitutions cause around 1.0∼1.5 kcal/mol reduction. As expected, only parts of R_1_-2 and D-1 residues are essential for Fep1 interaction. Leu524Ala at D-1 and Phe557Ala at R_1_-2 only reduce the binding free energies for 0.5∼0.8 kcal/mol. However, Tyr382 at R_1_-2 and Lys575 at D-1 are found to be required for binding Fep1, which decrease the binding affinity for around 1.7 and 1.9 kcal/mol, respectively. The energetic contributions are larger than R_1_ residues. As shown in Figure 12c, Tyr382 and Arg384 as well as Tyr508, Leu524, Phe557, Leu559 and Lys575 may form two binding clusters. The residues composed of binding cluster can stabilize each other through the interaction. It seems residues at the outer ring play major roles in pre-organizing the cluster. The Ala substitutions of Tyr382 and Lys575 may destroy the clusters. Thus, the ITC measurements of Tyr382Ala and Lys575Ala may reflect the net contribution of cluster collapse. This part will be reported in due time.

Although all measured values are less than 2 kcal/mol as the general definition of “hotspots”, we still consider the reduction of ∼1.0 kcal/mol binding free energy is enough to eliminate Tup11-Fep1 interaction. Leu559 at R_1_ in Tup11 from *S. japonicus* are at the same position as Leu542 from *S. pombe*. The single mutation of Leu542Ser destroys the interaction with Fep1 as detected by yeast two-hybrid method [38]. As shown in Figure 12b, Leu559Ala decreases the binding affinities for around 1.0 kcal/mol, suggesting that 1.0 kcal/mol is strong enough to eliminate the interaction in this specific case. Except for R_1_ residues, only part of R_1_-2 and D-1 are necessary for binding Fep1. This is because Tup11 may interact with multiple proteins on the top face, such as Tup1. Those residues, which are unimportant for interacting with Fep1, may be indispensable for the other proteins.

Conclusively, seven residues make the dominant contribution to the interaction as shown in **Figure 12c**. Compared with the previous achievements, five more residues are detected to be essential for interaction of Fep1 [38]. Thus, the feature-based method is capable of predicting hotspots for WD40 proteins from the primary sequences with a much higher reliability and efficient. By Figure 12c, we expect that Tup11-Fep1 interaction is dominated by hydrophobic interactions, which is currently under investigation.

Tup11 and their mutants have the similar CD spectroscopy at the same temperature and buffer as those in ITC reaction (**Figure S4**), which excludes the destruction of entire structure by Ala substitution. Thus, the changes of binding affinities are major due to the substitution effect. **Figure 12b** shows Arg382Ala, Lys575Ala and Ile601Ala have much larger error bars because the exothermic effects are dramatically decreased (**Figure S5**).

### Conclusion

An analysis of 36 crystal structures of WD40-repeat proteins reveals that WD_b–a_ bulge widely formed in strand b and strand a on the top surface. WD_b–a_ β-bulges have quite different residue propensities from those in the classic β-bulge as well as WD_c–d_. The X, R_1_ and R_2_ of the WD_b–a_ are dominated by small residues. Typically, these residues form inter-blade hydrogen bonds or salt-bridges to stabilize the proteins.

The R_1_ position in WD_b–a_ is also fairly populated with Arg, His, Ile, Leu, Lys, Met, Phe, Trp, Tyr and Val residues. All these residues adopt a protruded state on the top face due to the steric restraint, which have the abilities to provide highly energetic contribution in the protein-protein interaction. R_1_ as well as R_1_-2 are two residues close to the beginning of strand a, which are equal to the 18^th^ and 16^th^ residues proposed by Gaudet *et al* in Gβ-phosducin interaction [13]. Available crystallographic and mutagenesis studies of 29 protein complexes formed by 17 WD proteins support that R_1_-2, R_1_ and D-1 are generally required in protein-protein interactions.

With the use of the DHSW tetradsand β-bulges, the core sequence pattern has been obtained by aligning WD40 blades of 36 WD40 proteins. The resulting common sequence pattern provides clear information for the residue locations of the DHSW tetrad and WD_b–a_ β-bulge. Applying the dependency of R_1_-2, R_1_ and D-1 on the structural features, a feature-based method was developed to predict R_1_-2, R_1_ and D-1 from the primary sequences.

The predicted R_1_-2, R_1_ and D-1 by the feature-based method are consistent with the available mutagenesis studies of Met30, MDV1, COP1, SPA1 and Tup11. Twelve residues on R_1_-2, R_1_ and D-1 of Tup11 are predicted to be potential hotspots for recognizing Fep1. Five R_1_ (Arg384, Tyr426, Tyr508, Leu559, Ile601), one R_1_-2 (Tyr382) and one D-1 (Lys575) are convinced by ITC measurement to be important for binding Fep1. Compared with the previous result, the feature-based method is able to identify R_1_-2, R_1_ and D-1 precisely on the top face independent of crystal structure.

## Materials and Methods

### Protein Expression, Purification and Mutagenesis

The cDNA of *S. japonicus* Tup11 (WD40 domain: amino acids 301–630) and C-terminal region of *S. pombe* Fep1 (amino acids 319–564) were cloned into pET-28a (+) vectors. The alignment indicates WD40 domains of Tup11 from *S. japonicas* and *S. pombe* have the identical top face. All mutated DNA were prepared by following protocol of QuickChange site-directed mutagenesis kit. The constructs and mutants were confirmed by DNA sequencing. The wild type Tup11 and its mutants as well as Fep1 were overexpressed at 25°C in *E. coli* BL21 (DE3) with 0.1 mM IPTG. The proteins were purified by nickel affinity chromatography and followed by gel filtration. After the gel filtration, all proteins are finally dissolved in the 1×PBS buffer containing 0.01 mM Na_2_HPO_4_, 0.01 mM NaH_2_PO_4_, pH 7.4 and 150 mM NaCl. The Fep1 protein, Tup11 WT and its mutants were concentrated to ∼1.4 mM and ∼1 mM, respectively. The protein concentration was determined by NanoDrop 2000 Spectrophotometer (Thermo Scientific) as described in our previous work [56].

### ITC Measurement

The highly concentrated Fep1 was used directly, and Tup11 WT and its mutants were diluted to ∼0.2 mM by 1×PBS buffer in the titration. All ITC measurements were performed at 25°C on an iTC_200_ device (MicroCal). Total volume of 34 µl of Fep1 was injected by 2 µl each time at stirring speed of 1000 rpm, with 2 min injection spacing for equilibrium. The isotherm of Fep1 titrate into PBS buffer was set as background. Final data was obtained by subtracting background and fitting in Origin.

### CD Measurement

All CD measurements are carried out on a Chirascan Circular Dichroism (CD) Spectrometer (Applied Photophysics). Tup11 WT and its mutants were diluted to ∼0.1 mg/ml by deionized water, and were placed in a Hellma 1 mm light path Micro-cell separately. Three CD spectra were collected for each sample at 25°C over the wavelength range from 200 nm to 260 nm, with a 1 nm step wise. Three CD spectra of deionized water were averaged and set as baseline, and subtracted from the average spectrum of sample. At last, all CD data were converted to mean residue ellipticity.

## Supporting Information

Figure S1 **The intra−/inter-blade hydrogen bonds formed by two WD_b–a_.**
(TIF)

Figure S2 **The inter-blade salt bridges formed between Asp/Glu in the R_2_ of WD_b–a_ and Arg/Lys in the strand a of the other WD40 blades.**
(TIF)

Figure S3 **The prediction of R_1_, R_1_-2 and D-1 in the Tup11 from** ***S. pombe*** **, COP1 and SPA1 from** ***A. thaliana*** **.**
(TIF)

Figure S4 **CD profiles for Tup11 wild type and fourteen Ala substituted mutants.**
(TIF)

Figure S5 **ITC measurements of interaction between Tup11 substitutions and Fep1.**
(TIF)

Table S1 **The residues involved in 29 protein complexes formed by 17 WD proteins.**
(DOC)
