# Supplementary figures and images for: Identifying the Hotspots on the Top Faces of WD40-Repeat Proteins from Their Primary Sequences by β-Bulges and DHSW Tetrads
Source: PLoS One. 2012 Aug 15;7(8):e43005. doi: 10.1371/journal.pone.0043005 (PMC3419727; doi:10.1371/journal.pone.0043005)

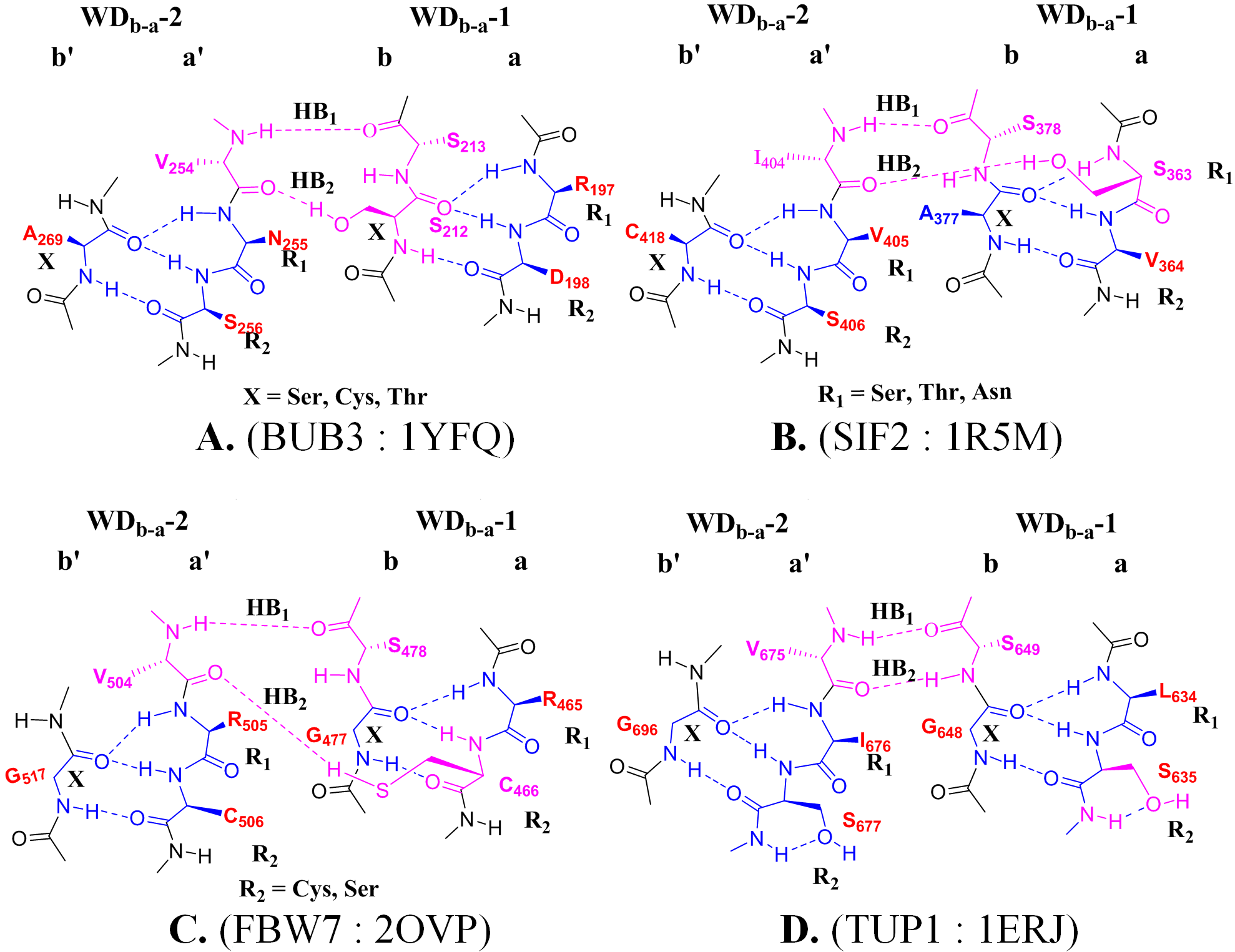

Supplement: Figure S1 — The intra−/inter-blade hydrogen bonds formed by two WDb–a. (TIF) [file pone.0043005.s001.tif]

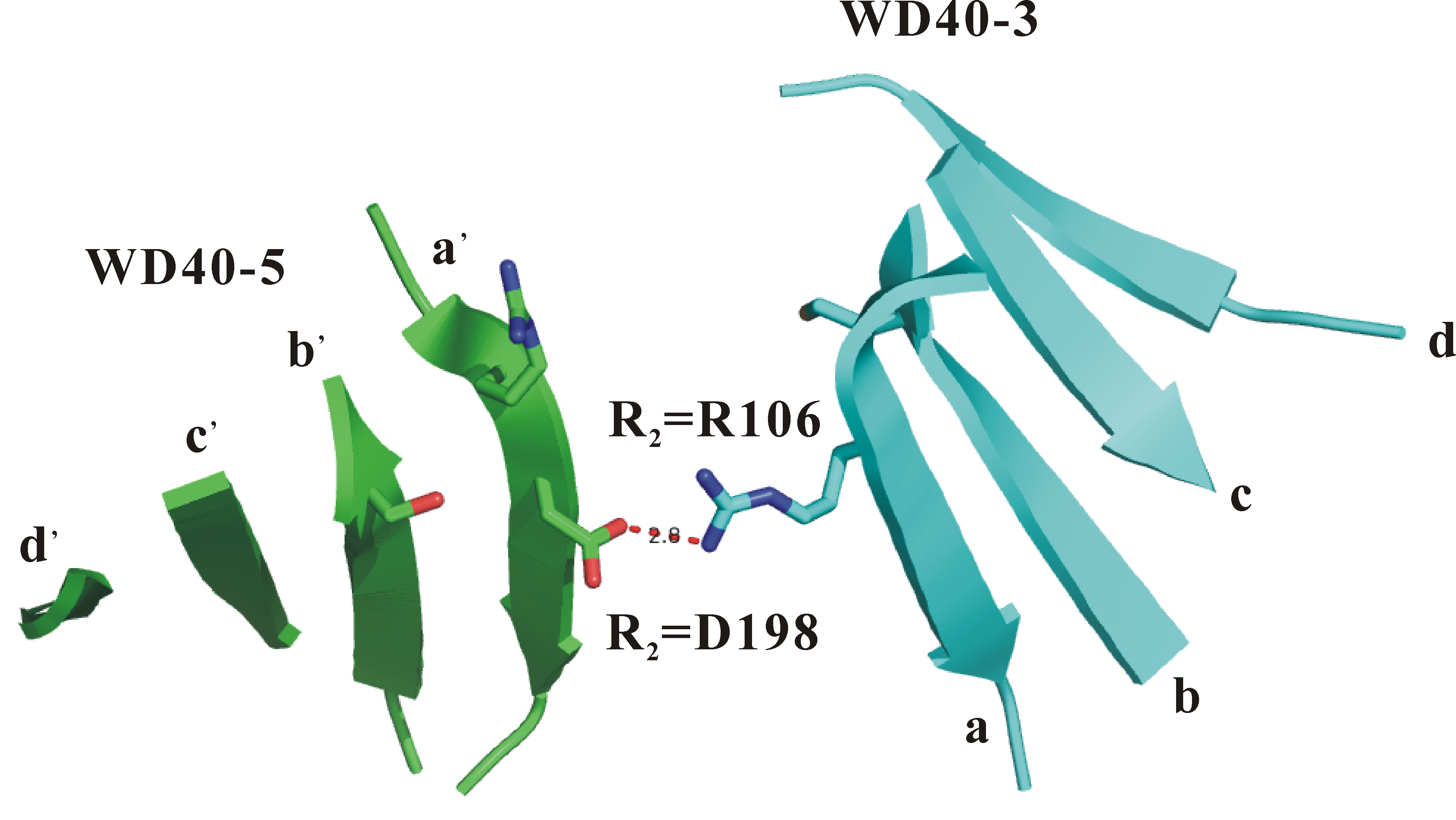

Supplement: Figure S2 — The inter-blade salt bridges formed between Asp/Glu in the R2 of WDb–a and Arg/Lys in the strand a of the other WD40 blades. (TIF) [file pone.0043005.s002.tif]

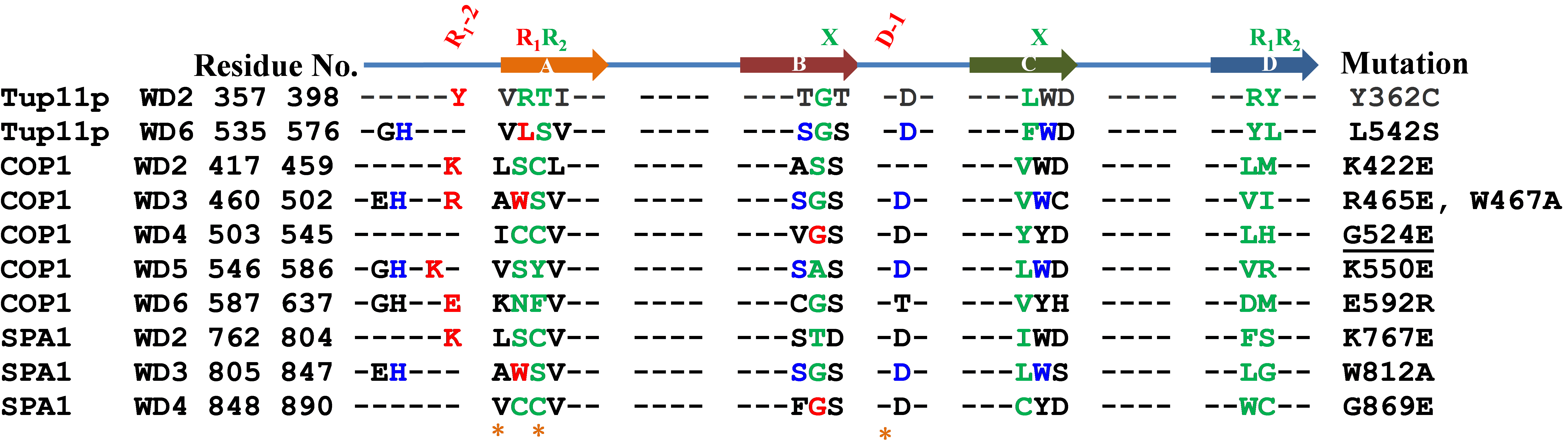

Supplement: Figure S3 — The prediction of R1, R1-2 and D-1 in the Tup11 from S. pombe , COP1 and SPA1 from A. thaliana . (TIF) [file pone.0043005.s003.tif]

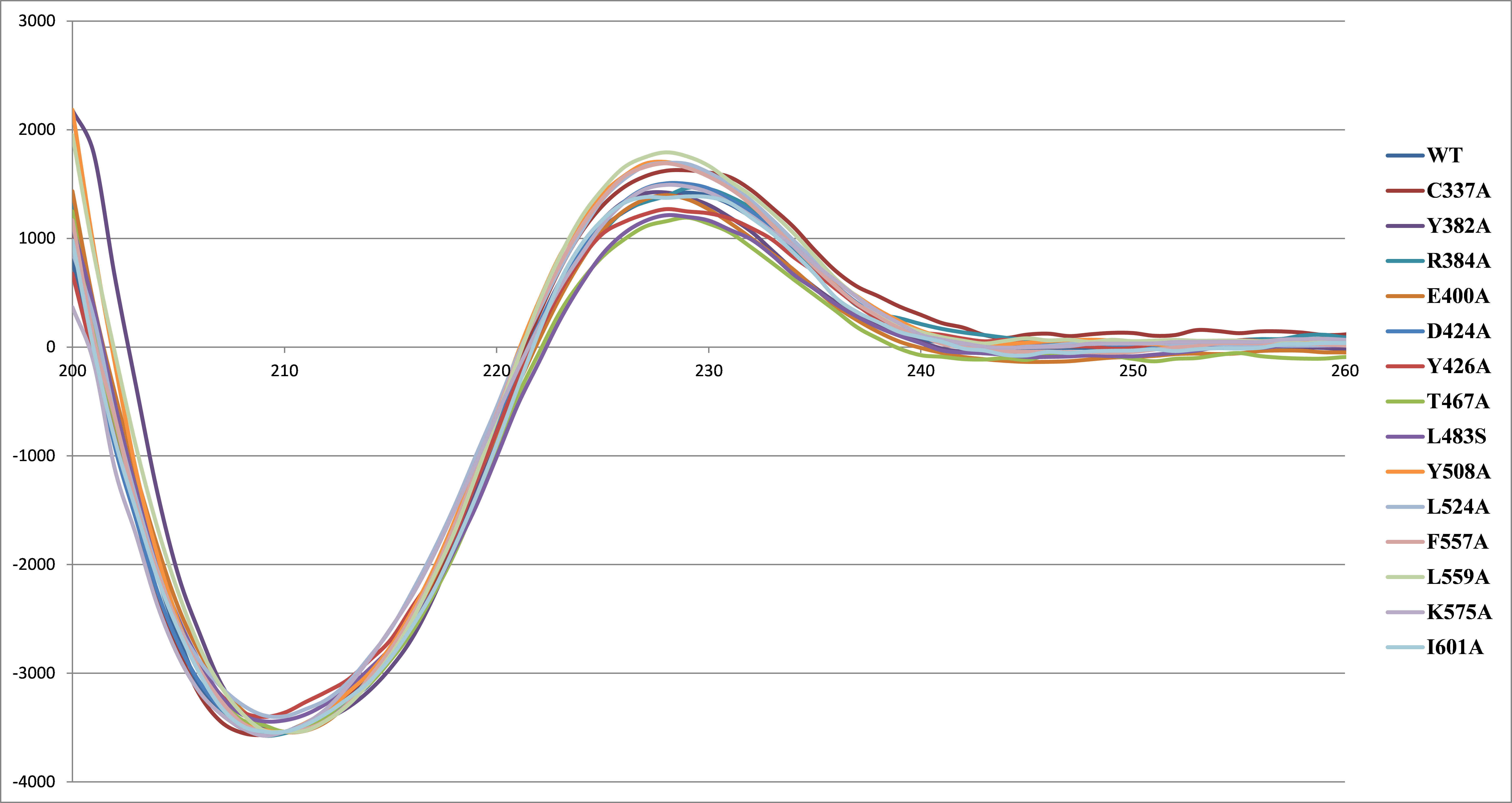

Supplement: Figure S4 — CD profiles for Tup11 wild type and fourteen Ala substituted mutants. (TIF) [file pone.0043005.s004.tif]
