# Supplementary material for: Identifying the Hotspots on the Top Faces of WD40-Repeat Proteins from Their Primary Sequences by β-Bulges and DHSW Tetrads
Source: PLoS One. 2012 Aug 15;7(8):e43005. doi: 10.1371/journal.pone.0043005 (PMC3419727; doi:10.1371/journal.pone.0043005)
Supplement: Table S1 — The residues involved in 29 protein complexes formed by 17 WD proteins. (DOC) [file pone.0043005.s006.doc]

**Table S1.** The protein complexes. The residues highlighted by underlines have further confirmed by the mutagenesis studies.

|  | **PDB_ID** | **Substrate** | **R_1_-2** | **R_1_** | **D-1** |
| --- | --- | --- | --- | --- | --- |
| **ARPC1 p40** | 1K8K | p20 | Q54, W204 | R100, L146, H206 | M350 |
| **Bub3** | 2I3S | Bub1 | Y13, L102, K152 | L60, F154, R197 | W31, V77, W120 |
|  | 2I3T | Mad3 | Y13, L102,K152, Q195 | L60, F154, R197 | W31, V77, W120, I214 |
| **β-TrCP1** | 1P22 | DSpGΦXSp | L472, R521 | Y271, L311, L351, N394, R474 | R285, K365, Y488 |
| **CDC4** | 1NEX | LLTpPP | R572 | W426, R467, R534,Y574, W717 | R485, Y548 |
|  |  | Ubiquitin^1^ | V384 | W717 | Y548 |
| **DOA1** | 3ODT | Ubiquitin^1^ | D15, F222 | W265 |  |
| **EED** | 3IIW | H3K27^3Me^ | Y308, *Y365* | F96, Y147, R414 |  |
| **FBW7** | 2OVQ | LPSGLLTpPPQSpG |  | W425, R465, R505, W673 | R479, Y519 |
|  | 2OVR | SLIPTpPDK | R689 | W425, R465, R505, W673 | R479, Y519, Y545 |
|  |  | Ubiquitin^1^ | L583 | Y545 |  |
| **Gβ** | 1OMW | GRK2 | K57, W99, Y145, R314 | Y59, M101, M188 | Q75, L117, D246, W332 |
|  | 2TRC | Phosducin | K57, W99, Y145, D186, D228 | Y59, M101, M188 | Q75, L117, D246, D290, W332 |
|  | 1XHM | SIGK peptide | K57, W99, Y145, D186, D228, R314 | Y59, M101, M188, N230 | L117, D246, W332 |
|  | 1GOT | Gα | K57, W99, Y145, D186, D228, R314 | Y59, M101, M188, N230 | Q75, L117, D246,W332 |
|  | 2PBI | RSG9 | K65, W107, Y155, D241, R327 | M109 | L125, D259 |
| **LIS1** | 1VYH | PAF-AFα2 | W340, F382 | R238 | R212, R316, D358 |
| **RAE1** | 3MMY | Nup98 | R172, Q214 | L90, Y174, R216 | W62, W149, I236 |
| **SEC13** | 3BG1 | Nup145C | M15, W214 | H17, W61, N107, R216, W266 | Y79 |
| **SEH1** | 3EWE | Nup85 |  | W308 |  |
| **Ski8p** | 1S4U | Ski3p^1^ | W293 | F20, W125, R237, F358 | F89, W311 |
| **Sec13** | 3JRP | Nup145C | W206 | H13, W57, R208, W258 | Y75, Q227 |
|  | 2PM9 | Sec31 | W206 | W57, R208, W258 | Y75 |
|  | 3MZK | Sec16 |  | H13, W258 |  |
| **TLE1** | 2CE9 | WRPW | Y532 | R534, Y578, F661, L702, L743 | E550, L636, K718 |
|  | 2CE8 | FxIxxIL | Y532 | R534,Y578, F661 | E550, L636, K718 |
| **TUP1** | 1ERJ | Matα2^1^ | Y445, N673 | Y580, Y489, L634, I676 | E463, K650 |
| **WDR5** | 2H13 | ARTKQ |  | F133, F263 | D107, F149, L321 |
|  | 3EMH | GSARAEVH |  | F133, F263 | D107, F149 |

Footnote. ^1^No crystal structure complex has been determined.
